# Introduction

**Published:** 1858-04

**Authors:** Jno. T. Toland


### THE DENTAL REPORTER.

VOL. 1. APRIL, 1858. No. 1.

### INTRODUCTION.

IN assuming, as I now do, the position of editor and publisher of a public Journal, it is due the profession, upon whom I must depend for encouragement and support, to make some explanation of my position, object, and intentions.

There are, doubtless, some who will deem this course of mine, as an act of *high presumption*, and will say, have we not already more Dental Periodicals than we want, even these are not supported, three have died a natural death within the past three years, and others are at the last gasp—albeit they are conducted by men of talents and scientific attainments—what then can this "*poor manufacturer* of *Dental Instruments*," and dealer in *Dentists Materials*, expect to accomplish. I will tell you! There is a wide field unexplored, and but slightly touched upon by the Journals of the day, which leaves a broken chasm through the impenetrable gloom of which the eye of the practical, hard working, and constantly occupied Dentist can not penetrate, because he can not spare time and money, to make primary, and thorough investigation of the WHOLE subject himself, and finding little assistance in the Literary of the profession, the objects, although of the first importance, and of the highest interests, still remain in calm repose.

*The first object*, is, the *production*, *cultivation* and *preservation* of *good teeth*.

In this, I think, all will agree with me, that the protection and preservation of the natural teeth, should be the *first* and *highest aim* of the *conscientious* and *scientific* dentist. Mechanical or artificial work comes in as secondary, and although the reputation and success of many of our most celebrated Dentists has grown out of their mechanical work, *they themselves* have more *pride* and self-satisfaction in the saving of one natural organ, which was supposed to have passed beyond hope (even though but poorly compensated), than in a whole denture of their most excellent productions ; but what of this, it is only "patch work" at best, the foundation is far beyond. We must begin with the fathers and the mothers of the generations yet unborn, both have their influence upon the constitution of the child; but as that of the mother is greatest, and from its longer continuance offers the best medium, the beginning must be made there.

Why is it, that a lady with beautiful and well developed teeth, will in a few months during the period of gestation, loose them, and rise from her confinement with pains and aches, teeth decaying and crumbling like ashes, a mouth full of snags! instead of the beauteous pearls that made her the envy and admiration of all who saw her before. Is there any other reason than that the appetite had been gratified beyond discretion, whilst nature was making large draughts upon the mother to supply materials for the bony tissue of the child, the food and medication of the mother was not such as to supply the elements to compensate for this loss.

How is it with the child. If the materials for the formation of bone, were deficient in the mother, the bony structure of the child must be defective. It is born and fed with the milk insufficiently supplied, its teeth make their appearance, and almost as soon as they are through the gum, they begin to decay, and are lost before the permanent organs have time to be developed, the consequence is a contraction of the arch ill-formed, irregular, and imperfectly developed teeth, which soon decay in turn, the result is that it is almost impossible to find a person of thirty years with a sound set of teeth, there is no reason (accidents excepted,) why the teeth should not last as long as the remainder of the body—it should be corrected. This is a field in which *dentists*, *physicians*, *chemists*, *naturalists*, and the *public* can co-operate in throwing light on this subject, and we will be pleased to receive communications for publication from all.

The SECOND object is. To elevate the *profession* not only intrinsically and in the estimation of its members, but also in the minds of the *people*, to explain to the public, the great importance of the dental organism, its effects upon, not only the appearance, expression, and voice, but also upon the *general health*, the importance of *early* and proper attention to the teeth, and the still greater importance of having dental operations performed well, regardless of the expense, to convey to the people the great truth, that the health and preservation of the teeth is of vital importance; being so, the first consideration should be to have operations performed in the best possible manner, that to have dental operations performed cheap, unless they are *well done* is no object; but that the first consideration should be, to have their teeth attended to in the best possible manner, that if it is worth doing at all, it is worth doing well, and if not well done, it were better not done at all; that in order to have the time, care and attention bestowed, which will enable the dentist to do the best work he can, he must be well paid, that it is cheaper to preserve the natural organs in health and beauty, than to have all the artificial work that can be made; but, that whatever is to be done, whether operative or mechanical, should be done in the highest state of perfection, that instead of beating down in price, and endeavoring to see how cheap they can have it done, they should endeavor to see how perfectly it can be accomplished. Under the present system, a large proportion of dental operations are worthless, because the patients are unwilling to pay a compensating price for the time and labor, to do the most perfect work, to effect this, the influence must be brought to bear upon the patient direct, and upon the family physician; and therefore, the "Reporter" should be made to circulate among the physicians, and the public as well as the dentists. Several with whom we have conversed on this subject, say that if they can not induce physicians and patients to subscribe for the work, they will rather take a dozen numbers themselves and distribute them gratuitously.

The third object is. To give prompt and reliable information on dental improvements and inventions. Placing in reach of our subscribers the information and means of knowing and experimenting on all new processes, before spending their money for something which may prove worthless. We do not believe in the principle of buying a "pig in a poke." And it is believed, that with the assistance of the profession, even the attempt to crowd upon them the humbug vagaries, which are daily springing up, untried, will cease.

An intelligent and liberal profession should be allowed to use their knowledge and judgment, instead of being placed in a position where they must either not use nor even search into the "hidden mystery," unless they will purchase the lawful privilege, "Sight unseen."

During the past two years, I have printed and distributed among the profession in six different issues, about 25,000 pamphlets, containing from 32 to 50 pages; these, I sent out, paid postage, &c., all free, at an aggregate cost of about $2,500, because I used a portion of the space for the advertisements of my business, this has now been sufficiently effected. In the various issues of the "Reporter," I have in a meagre way, thrown light upon the hidden things, of the mystery loving portion of the profession. What I have thus done was practical, not theoretic, and I think I may safely say, without boasting or the fear of contradiction, that I have thus saved to the *pockets* of the practising dentists of the country, at least as many thousands as I have spent hundreds in doing it. And now I think it but fair that whilst I give them such information as they can get through no other channel, they should pay the expense, to each of them it is but a trifle, to me it is a large amount, and if the invention and patenting mania continues, as it has for a few years past, there will still be ample opportunity for labor and exposure, and to give you at least the *worth* of *your money.*

I have no hesitation in saying, that whoever subscribes for the Reporter, and at the end of the year will say, that he is not fully compensated, I will cheerfully refund his money.

The size of the Reporter, will be for the present, from 48 to 64 pages. I intend that both in *quantity* and *quality* of matter, it shall occupy the highest point, which the liberality of subscriptions will justify, if the subscriptions will pay the *expenses*, we will be satisfied, if 2000 subscribers can be obtained, we can afford at the same price, twice as good a publication as we could if only 500 are taken, because the expense of printing 2000 copies is only about double what 500 would cost.

It is estimated that there are about 7000 dentists in the United States, and 3000 of them in the west and south-west, of which Cincinnati is the centre. One dollar a year to the dentist is a small investment, if he can save or make thereby $25, $50 or $100, or even more.

The "Reporter" will not be *confined* to the objects above named, but will take free and independent scope, touching upon every thing of interest to the profession, mechanical, operative, literary, and scientific.

Our style is *our own*, and shall to the best of our ability be plain and practical, avoiding a fine spun array of words, which too frequently mean nothing! We care not even to be strictly grammatical, so that we can in a brief, plain, common sense way, bring the important facts to the comprehension of our friends, we will deem our duty accomplished.

Those who may feel inclined to revise or criticise, may, therefore, save themselves the trouble, as we do not intend that our "feelinks" shall be seriously wounded by such.

JNO. T. TOLAND.

